# Age, Body Mass Index, and Number of Previous Trials: Are They Prognosticators of Intra-Uterine-Insemination for Infertility Treatment? 

**Published:** 2014-11-01

**Authors:** Ahmed M. Isa, Basim Abu-Rafea, Saleh A. Alasiri, Saleh Binsaleh, Kareema H. Ismail, George A. Vilos

**Affiliations:** 1 Department of Obstetrics and Gynecology, Assisted Conception Unit, College of Medicine, King Saud University, Riyadh, Saudi Arabia; 2 Department of Surgery, Division of Urology, College of Medicine, King Saud University, Riyadh, Saudi Arabia; 3 Western Ontario University, London, Ontario, Canada

**Keywords:** Intrauterine Insemination, Age, Body Mass Index, Pregnancy Rate

## Abstract

**Background:**

To examine whether pregnancy rate (PR) of intrauterine insemination (IUI) is related to certain demographic factors, such as age and body mass index (BMI), along with number of IUI cycles performed, a set of infertile Saudi women.

**Materials and Methods:**

During this prospective study (a 24-month period), 301 Saudi women with infertility underwent IUI in our infertility clinic. We investigated whether PR is correlated with patient age and BMI, and the number of IUI trials, in order to determine if they could be used as prognosticators of pregnancy success.

**Results:**

The highest PR was 14.89% for ages 19-25 and the lowest PR was 4.16% for ages 41-45, indicating no statistically significant difference among PR in all age groups (p value of 0.225). Also, in terms of BMI, the highest PR was 13.04% for BMI ≥35 and the lowest was 7.84% for BMI of <25 to 18.5, indicating no significant difference among different BMI groups (p value of 0.788). One-cycle treatment, as expected, was more successful (PR=12.84%) than 2-cycle treatment (PR=5.75%), however, 3-5-cycles treatment still showed encouraging results (PR=17.24%); but the difference did not reach statistical significance (p value=0.167).

**Conclusion:**

PR after IUI treatment remained approximately 10% from 19 to 40 years of age and declined after 40. Although no significant difference was observed among different age groups, earlier treatment is still recommended. There was a positive but not statistically significant correlation between PR and patient’s BMI indicating that BMI is not a determining factor. There was also no correlation between PR and number of IUI trials. Patients can thus try as many times as they want before moving on to *in vitro* fertilization (IVF) treatment.

## Introduction

The advantages of intrauterine-insemination (IUI) with and without mild ovarian stimulation were early recognized and IUI was applied for treating couples with borderline male, cervical, immunological, or unexplained infertility factors (1). Additionally, IUI has been shown to be a much less expensive and less invasive procedure in comparison with the *in vitro* fertilization-embryo transfer (IVF-ET) (2).

Following IUI, pregnancy rates vary widely due to multiple factors including heterogeneity and variability of studied patients and parameters (3- 7). These variations could also be due to population differences and circumstantial variability under which each study was conducted (1).

The objective of the present study is to audit the clinical outcomes of IUI as a mild infertility treatment in a set of female patients in Saudi Arabia and to examine whether clinical outcomes were related to patient demographic factors such as age and BMI, as well as, the number of IUI treatments, in order to be used as success prediction factors.

## Materials and Methods

During a 24-month period, between January 2010 and December 2011 inclusive, 301 Saudi women with infertility underwent extensive investigation consecutively. That included pertinent infertility-related history, general physical and pelvic examination, and assessment of the reproductive organs by appropriate imaging and endoscopy. Baseline serum levels of follicular stimulating hormone (FSH), luteinizing hormone (LH), thyroid stimulating hormone, and prolactin were always analyzed. In addition, assessment of the male partner included at least two semen analyses, with at least one analysis being performed before and after sperm wash to determine the total motile sperm count and modes of sperm progression. Further evaluation of the male partner included analysis of serum levels of FSH and testosterone, when indicated. Eligible women for IUI-treatment were then stimulated with different kinds of ovulation induction hormones. Most patients were stimulated with highly purified gonadotropins (hMG), such as Merional, 75 i.u./day or Puregon, 50 i.u./day (both from IBSA, Switzerland). Only cases that did not show any sign of response within the first ten days of treatment were excluded. The number of treatment days differed based on various factors, like age, body mass index (BMI), and cause and history of infertility. A number of patients were stimulated with clomiphene citrate, 100 mg/day (Merk, Germany) for 5-6 days. All patients were monitored with ultrasound for ovulation induction and follicle maturation. When a maximum of two leading follicles reached at least 17 mm in diameter, patients were injected with 5,000 IU of human chorionic gonadotropin hormone (HCG) 36 hours prior to IUI.

On the day of IUI, fresh semen of the husband was prepared for insemination. The final sperm specimen was mixed in 0.5 ml of HEPES-buffered media (Quinn’s Advantage Medium, with HEPES, SAGE (Pasadena, USA), supplemented with serum (Quinn’s Advantage Serum Protein Substitute SPS, SAGE (Pasadena, USA). All pre- and postpreparation semen parameters were recorded.

Pregnancy was determined by βHCG values obtained on the sixteenth day after IUI, and then confirmed by ultrasound four weeks later.

### Statistical analysis

Data were analyzed using the SPSS statistical software package, version 19 (SPSS Inc., Chicago, USA). Chi-squared test was used to compare pregnancy rates with respect to all variables. P-value less than 0.05 were considered statistically significant.

### Ethical considerations

The Institutional review board of the College of Medicine at King Saud University approved this study with approval number E-12-642. Informed written consents were also obtained from all human adult participants in the study.

## Results

Women were stratified by age as shown in table 1. The median age of the entire group was 31, with a range of 19-45. The highest pregnancy rate was 14.89% in age group 19-25, as compared with the lowest PR of 4.16% in age group 41-45. This difference was not statistically significant (p=0.225). One pregnancy occurred, at age 44, among 24 women over 41 years.

BMI was stratified according to the World Health Organization (WHO) definition (8, 9). The relationship between BMI and pregnancy rates among all groups is shown in table 2. The median BMI of the group was 30.14. The highest PR was 13.04% with BMI ≥35, while the lowest PR was 7.84% with BMI from 18.5 to less than 25. This difference was also not statistically different (p=0.788).

The relationship between the number of IUI cycles performed and pregnancy rates is shown in table 3. The differences between one, two, and three to five cycles did not reach statistical significance (p=0.167), however, there was a trend that suggested the first treatment cycle to be the most successful.

**Table 1:** Relationship between age and pregnancy rate of infertile women treated with intrauterine insemination


	Age (Y)	Number of patients and (%)	Number of pregnancies and (PR%)	P value/χ^2^

**I.**	**19-25**	47(15.51%)	7(14.89%)	
**II.**	**26-30**	106 (34.98%)	6(5.66%)	
**III.**	**31-35**	81(26.73%)	11 (13.58%)	0.225
**IV.**	**36-40**	43(14.19%)	5(11.63%)	χ^2=5.673^
**V.**	4**1-45**	24(7.92%)	1(4.16%)	
		**Total: 301**	**Overall PR: ~10%**	


PR; pregnancy rate.

**Table 2:** Relationship between body mass index (BMI), and pregnancy rate of infertile women treated with intrauterine-insemination


	BMI (Kg/m^2^)	Number of patients and (%)	Number of pregnancies and (PR)	P value/χ^2^

**I.**	**Normal (18.5-<25)**	51 (16.94%)	4(7.84%)	
**II.**	**Over-Weight (25-<30)**	97 (32.23%)	9(9.28%)	0.788
**III.**	**Obese (30-<35)**	84 (27.91%)	8(9.52%)	χ^2=1.054^
**IV.**	**Highly Obese (≥35)**	69 (22.92%)	9(13.04%)	


PR; Pregnancy rate and BMI; Body mass index.

**Table 3:** Relationship between Number of intra-uterine insemination cycles performed and pregnancy rate in saudi infertile women


	Number of IUI Cycles	Number of patients and (%)	Number of pregnancies and (PR)	P value/χ^2^

**I.**	One	148 (49.17%)	19 (12.84%)	0.167
**II.**	Two	87 (29.04%)	5(5.75%)	χ^2=3.576^
**III.**	3-5	66 (21.93%)	5(17.24%)	


PR; Pregnancy rate and IUI; Intra-uterine insemination.

## Discussion

We audited results from a single infertility clinic in the Kingdom of Saudi Arabia, to determine whether women’s age, and BMI, as well as, the number of IUI treatments performed could be used as prognostic indicators of pregnancy success.

Data in table 1 shows relatively good outcome up to the age of 40, while some studies reported good outcomes only up to 30 years of age, (10-12) and reporting favorable outcomes up to age 35 (13-15). Although there was a decline by approximately 50% in PR in the 26-30 age group, the difference was not statistically significant. There may be a logical explanation related to local socio-demographic factors. Women of 26-30 years of age comprise the most active reproductive group as they are either recently or just married and are actively trying to conceive. Therefore, those who fail to spontaneously conceive may have more complicated infertility issues, and IUI may not be the best treatment for them. With the exception of the 26-30 age group, the results were in line with those published by others (11).

The lowest pregnancy rate (4.16%) was in the over-40 group (one success out of 24 cycles). These findings are in agreement with those of others (10). One study (16) reported PR of only 2% for 40 years old or older patients. Therefore, many authors advised women of that age not to undergo IUI more than once. Moreover, one study (14) reported no pregnancy after age 40, while another (17) reported no pregnancy after age 44. Consequently, it was advised that infertile women of 43 or older seek directly other alternatives (18).

We found that normal BMI was found in only 16.8% of women while 27.8% were highly obese. These statistics are very disturbing from a public health point of view as this group of patients is a random sample of Saudi women. It has been proven that overweight/obesity has become a world epidemic, (19) that is associated with multiple health issues including pregnancy related complications (20). Obesity is related to ovulation dysfunction that leads to limited fertilization, and menstrual disorders, which may lead to impaired implantation (21-23). Ovulation interruptions are known to be more related to polycystic ovarian syndrome which is more common in obese women. The incidence of intrauterine lesions such as myomas and/or polyps is also more frequent in obese women (24, 25). Other complications during and after birth (19, 20) will not be discussed as they do not pertain to this study.

We found, however, no statistically significant difference in PR between different BMI groups, including the highly obese group (BMI ≥35). A study (26) reported that ovulation induction with an aromatase inhibitor (letrozole 2.5 mg for 5 days) and IUI treatment, showed no significant difference for different BMI’s. Another study (27) reported that obese women produce fewer follicles and require higher doses of medication; the success rate however was comparable to that of women with normal weight.

On the other hand, some studies (23, 24, 28) reported that overweight and obese sub-fertile women had a reduced probability of successful fertility treatment and their pregnancies were associated with more complications and higher costs. Few other studies (29-31) have also reported that weight loss helped the ovulation process in some obese women and enhanced their infertility treatment outcome.

About half (49.17%) of the patients underwent IUI only once, with PR of 12.84%. Then the PR declined to 5.75% with 29.04% of the patients who tried IUI twice. The PR then climbed up again to 17.24% with those who underwent 3-5 IUI cycles (21.93% of the patients), although with no significant difference among all three groups. A study (32) compared PR from one to six IUI cycles, and found that the PR of the first cycle is significantly higher than the rest of the IUI cycles. A similar study (14) found that the PR rate increased up to the third trial and decreased thereafter, contrary to a study (13) that found IUI pregnancy rates were significantly higher after the third cycle. Another study (33) found no superiority of a specific cycle which is more consisted with our findings. Campana et al. (17) found significantly higher cumulative pregnancy rates with up to six IUI’s compared with those with up to three IUI. Botchan et al. (34) reported pregnancies at the eighteenth IUI cycle (one pregnancy out of eighteen patients) and above, (five pregnancies out of ninety-five cases). So, it may be advisable that couples who have concerns about their time or finances, be counseled to proceed with IVF-ET if their first IUI trial has failed.

## Conclusion

We audited a group of 301 infertile Saudi women treated with IUI and found that the pregnancy rate remained approximately 10% for age groups between 19 and 40. While after 40 it declined noticeably, which means that direct IVF treatment would be in their favor. There was a positive but not statistically significant correlation of pregnancy rates with BMI; (from 8% PR, for normal BMI, to 13%, for highly obese), i.e. female high BMI is not a discouraging factor for IUI treatment. There was also no clear correlation between PR and the number of IUI trial; and it is therefore the patient’s choice when to move to IVF treatment instead.

## References

[1] Iberico G, Vioque J, Ariza N, Lozano JM, Roca M, Llacer J (2004). Analysis of factors influencing pregnancy rates in homologous intrauterine insemination. Fertil Steril.

[2] Kossakowski J, Stephenson M, Smith H (1993). Intrauterine insemination with husband’s sperm: comparison of pregnancy rates in couples with cervical factor, male factor, immunological factor and idiopathic infertility. Aust N Z J Obstet Gynaecol.

[3] Allen NC, Herbert CM 3rd, Maxson WS, Rogers BJ, Diamond MP, Wentz AC (1985). Intrauterine insemination: a critical review. Fertil Steril.

[4] Nuojua-Huttunen S, Tomas C, Bloigu R, Tuomivaara L, Martikainen H (1999). Intrauterine insemination treatment in subfertility: an analysis of factors affecting outcome. Hum Reprod.

[5] Gezginc K, Gorkemli H, Celik C, Karatayli R, Ciçek MN, Olakoglu MC (2008). Comparison of single versus double intrauterine insemination. Taiwan J Obstet Gynecol.

[6] Demirol A, Gurgan T (2007). Comparison of different gonadotrophin preparations in intrauterine insemination cycles for the treatment of unexplained infertility: a prospective, randomized study. Hum Reprod.

[7] Freour T, Jean M, Mirallie S, Langlois ML, Dubourdieu S, Barriere P (2009). Predictive value of CASA parameters in IUI with frozen donor sperm. Int J Androl.

[8] World Health Organization (1995). Physical status :the use and interpretation of anthropometry.Report of WHO Expert Committee. World Health Organ Tech Rep Ser.

[9] World Health Organization (2000). Obesity: preventing and managing the global epidemic.Report of a WHO consultation.

[10] Cruz JR, Gindoff PR (1999). Age and reproduction. Reproductive Medicine Review.

[11] Demir B, Dilbaz B, Cinar O, Karadag B, Tasci Y, Kocak M (2011). Factors affecting pregnancy outcome of intrauterine insemination cycles in couples with favorable female characteristics. J Obstet Gynaecol.

[12] Merviel P, Heraud MH, Grenier N, Lourdel E, Sanguinet P, Copin H (2010). Predictive factors for pregnancy after intrauterine insemination (IUI): an analysis of 1038 cycles and a review of the literature. Fertil Steril.

[13] Yang JH, Wu MY, Chao KH, Chen SU, Ho HN, Yang YS (1998). Controlled ovarian hyperstimulation and intrauterine insemination in sub-fertility.How many treatment cycles are sufficient?. J Reprod Med.

[14] Zadehmodarres S, Oladi B, Saeedi S, Jahed F, Ashraf H (2009). Intrauterine insemination with husband semen: an evaluation of pregnancy rate and factors affecting outcome. J Assist Reprod Genet.

[15] Kamath MS, Bhave P, Aleyamma T, Nair R, Chandy A, Mangalaraj AM (2010). Predictive factors for pregnancy after intrauterine insemination: a prospective study of factors affecting outcome. J Hum Reprod Sci.

[16] Harris ID, Missmer SA, Hornstein MD (2010). Poor success of gonadotropin-induced controlled ovarian hyperstimulation and intrauterine insemination for older women. Fertil Steril.

[17] Campana A, Sakkas D, Stalberg A, Bianchi PG, Comte I, Pache T (1996). Intrauterine insemination: evaluation of the results according to the woman’s age, sperm quality, total sperm count per insemination and life table analysis. Hum Reprod.

[18] Corsan G, Trias A, Trout S, Kemmann E (1996). Ovulation induction combined with intrauterine insemination in women 40 years of age and older: is it worthwhile?. Hum Reprod.

[19] Aviram A, Hod M, Yogev Y (2011). Maternal obesity: implications for pregnancy outcome and long-term risks-a link to maternal nutrition. Int J Gynaecol Obstet.

[20] Djelantik AA, Kunst AE, Van Der Wal MF, Smit HA, Vrijkotte TG (2012). Contribution of overweight and obesity to the occurrence of adverse pregnancy outcomes in a multi-ethnic cohort: population attributive fractions for Amsterdam. BJOG.

[21] Teede H, Deeks A, Moran L (2010). Polycystic ovary syndrome: a complex condition with psychological, reproductive and metabolic manifestations that impacts on health across the lifespan. BMC Med.

[22] Motta AB (2012). The role of obesity in the development of polycystic ovary syndrome. Curr Pharm Des.

[23] Yogev Y, Catalano PM (2009). Pregnancy and obesity. Obstet Gynecol Clin North Am.

[24] Al-Nuaim LA (2011). The impact of obesity on reproduction in women. Saudi Med J.

[25] Nappi L, Indraccolo U, Di Spiezio Sardo A, Gentile G, Palombino K, Castaldi MA (2009). Are diabetes, hypertension, and obesity independent risk factors for endometrial polyps?. J Minim Invasive Gynecol.

[26] McKnight KK, Nodler JL, Cooper JJ Jr, Chapman VR, Cliver SP, Bates GW Jr (2011). Body mass index-associated differences in response to ovulation induction with letrozole. Fertil Steril.

[27] Souter I, Baltagi LM, Kuleta D, Meeker JD, Petrozza JC (2011). Women, weight, and fertility: the effect of body mass index on the outcome of superovulation/intrauterine insemination cycles. Fertil Steril.

[28] Koning AM, Kuchenbecker WK, Groen H, Hoek A, Land JA, Khan KS (2010). Economic consequences of overweight and obesity in infertility: a framework for evaluating the costs and outcomes of fertility care. Hum Reprod Update.

[29] Al-Azemi M, Omu FE, Omu AE (2004). The effect of obesity on the outcome of infertility management in women with polycystic ovary syndrome. Arch Gynecol Obstet.

[30] Clark AM, Ledger W, Galletly C, Tomlinson L, Blaney F, Wang X (1995). Weight loss results in significant improvement in pregnancy and ovulation rates in anovulatory obese women. Hum Reprod.

[31] Clark AM, Thornley B, Tomlinson L, Galletley C, Norman RJ (1998). Weight loss in obese infertile women results in improvement in reproductive outcome for all forms of fertility treatment. Hum Reprod.

[32] Khalil MR, Rasmussen PE, Erb K, Laursen SB, Rex S, Westergaard LG (2001). Homologous intrauterine insemination.An evaluation of prognostic factors based on a review of 2473 cycles. Acta Obstet Gynecol Scand.

[33] Zhao Y, Vlahos N, Wyncott D, Petrella C, Garcia J, Zacur H (2004). (2004) Impact of semen characteristics on the success of intrauterine insemination. J Assist Reprod Genet.

[34] Botchan A, Hauser R, Gamzu R, Yogev L, Paz G, Yavetz H (2011). Results of 6139 artificial insemination cycles with donor spermatozoa. Hum Reprod.

